# Exploring the contextual transition from spinal cord injury rehabilitation to the home environment: a qualitative study

**DOI:** 10.1038/s41393-020-00608-y

**Published:** 2021-02-09

**Authors:** Lene Weber, Nanna Hoffgaard Voldsgaard, Nicolaj Jersild Holm, Lone Helle Schou, Fin Biering-Sørensen, Tom Møller

**Affiliations:** 1 The University Hospitals Centre for Health Research (UCSF), Rigshospitalet, Copenhagen University Hospital, Department, 9701 Copenhagen, Denmark; 2 Research Unit on Brain Injury Rehabilitation Copenhagen (RUBRIC), Department of Neurorehabilitation, Traumatic Brain Injury Unit, Rigshospitalet, Copenhagen University Hospital, Copenhagen, Denmark; 3 Department of Spinal Cord Injuries, Rigshospitalet, Copenhagen University Hospital, Copenhagen, Denmark; 4 Department of Nursing and Nutrition, University College Copenhagen, Copenhagen, Denmark

**Keywords:** Spinal cord diseases, Quality of life

## Abstract

**Study design:**

Explorative qualitative study based on an interpretative phenomenological approach.

**Objectives:**

This study explored the possibility of transferring knowledge and skills from a spinal cord injury (SCI) unit to the home environment; the individual and structural factors that potentially influenced this transfer; and its compatibility with a meaningful everyday life.

**Setting:**

Hospital-based rehabilitation unit and community in Denmark.

**Methods:**

Fourteen individuals with SCI were selected with maximum variation according to age, sex, marital status, and level of injury. In-depth, semi-structured interviews were conducted in the participants’ homes, 2–10 months after discharge from an SCI unit. Data analysis involved taking an interpretative phenomenological approach combined with a template analysis and applying the transfer of training theory to the discussion.

**Results:**

Transitioning from the SCI unit to the home environment involved a multidimensional change of context in which most of the participants’ previous life roles had changed. This overarching theme had a decisive influence on: balancing loss and acceptance, facing external structural barriers, and the strength of social relationships when the knowledge and skills acquired at the unit were applied in a meaningful everyday life.

**Conclusions:**

Transition from the SCI unit to the home environment is influenced by a multidimensional change of context that may restrict the use of acquired skills post-discharge, provide distant prospects for tertiary health promotion, and aggravate the experience of loss in people with SCI. Maintaining relationships is a strong mediator for transferring skills and re-establishing a meaningful everyday life.

## Introduction

Individuals who sustain a spinal cord injury (SCI) may experience severe functional and long-lasting impairments, such as loss of locomotor function, pain, spasticity, bladder/bowel, and sexual dysfunction [1, 2]. A meta-analysis concluded that, in addition to the physical consequences, depression was reported in 22% of individuals with SCI [3]. Another prospective study found that 17–25% experience increased psychosocial distress following SCI and after transitioning into the community [4]. Lower quality of life is particularly evident in the early post-injury years [5].

Organizing SCI treatment into specialized interdisciplinary care centers has led to significant improvements in major SCI outcomes [6], reducing mortality and the number and severity of SCI complications [7] and increasing the likelihood of being discharged to the home [8]. Recently, clinical practice guidelines were established for identifying and managing cardiac metabolic risk following SCI [9]. Some SCI units now offer a teaching program to facilitate an active, healthy lifestyle post-discharge to prevent long-term lifestyle complications [10, 11]. In Denmark, ~95% of individuals with SCI are discharged to their home environment [12]. Following discharge municipal authorities are expected to bear the financial responsibility for providing equipment, continued care, and rehabilitation.

Relatively few qualitative studies have focused on the transition from the SCI unit to the immediate post-discharge period [13–15]. Findings suggest that everyday life is burdened by a demanding body, challenges in acquiring assistance needed at home, and barriers to returning to the wider community. Transfer of training, a theoretical perspective derived from organizational and educational research, represents a way to shed more light on transitional changes at the individual level [16, 17]. This perspective refers to the application, generalization, and maintenance of trained skills during contextual changes and recognizes the gap that exists between the acquisition effort and the application outcome [17]. Does a similar gap exist in SCI rehabilitation? Skills taught at the SCI unit are implicitly assumed to be transferable to the home environment, but the factors that allow people with SCI to manage this transfer of knowledge and skills are unclear.

Consequently, this qualitative study emphasizes the individual’s perspectives on transition. The study aims to explore the possibility of transferring knowledge and skills from the SCI unit to the home environment; the individual and structural factors that potentially influence this transfer; and its compatibility with a meaningful everyday life.

## Methods

### Design

This is a qualitative study that uses an interpretative phenomenological analysis (IPA) approach [18] to assess empirical data generated from in-depth, semi-structured interviews of individuals with SCI following specialized rehabilitation in Denmark. IPA draws upon phenomenology, hermeneutics, and idiography [18].

### Participant recruitment

The underlying principles behind sufficient “information power” [19] guided the researchers to an adequate sample size of 12–14 participants recruited from the Department of Spinal Cord Injuries in Eastern Denmark. The primary researchers (LW and NHV) collaborated with a clinical specialist (NJH) from the SCI unit to identify potential participants from an existing cohort of individuals with SCI [11]. Inclusion criteria were as follows: injured with SCI within the last 12 months at admission to rehabilitation, regardless of SCI etiology, neurological level, or completeness of the lesion; >18 years of age; Danish speaking; discharged to home within 2–10 months at the time of the interview. People were not recruited if they suffered from a psychotic disorder. Purposive sampling was used to select participants based on achieving maximum variation according to age, sex, marital status, and level of injury [19]. LW or NHV contacted the participants to provide information on the study, collect information on living arrangements, schedule an interview, and obtain written consent.

### Data collection

Interviews, conducted from January 2019 to April 2019, took place in the participants’ homes to encourage open conversation and to increase access to the context in which the application of skills unfolded.

The semi-structured interview guide was developed based on a search of the literature on transition, theoretical transfer of training perspectives, and discussions with research colleagues (FBS, NJH, and LHS). Three broad a priori themes (individual story, experience of rehabilitation, and experience of everyday life) provided the underlying foundation of the guide, which nonetheless allowed ample space for participants’ individual narratives and exploration of new, unanticipated topics. Supplementary online material (S1) contains the initial template for the interview guide. Interview questions followed Miller and Crabtree’s recommendations for in-depth interviews [20]. To ensure credibility, respondent validation was used by carefully summarizing participants’ descriptions during the interview. The final questions varied, depending on the individual narrative, but an effort was made to conclude in a positive atmosphere. Fieldnotes and the researchers’ initial reflections were documented during each interview. All interviews were audio recorded and transcribed verbatim, with selected quotes translated into English by a native speaker.

When researchers commence a study, their personal, professional, and theoretical background influences all stages of their investigation [21]. The primary researchers, LW and NHV, are both physiotherapists, have an MSc in health science, and more than 10 years of neurological rehabilitation experience but did not take part in the participants’ SCI rehabilitation. TM is a nurse, Ph.D. and a senior researcher who has executed numerous qualitative studies.

### Analysis

Two complementary methods [22], IPA [18] and template analysis [23], guided analysis of the participants’ rich narratives and formed the theoretical foundation of the transfer of training. In accordance with Smith et al. [18], we used a six-step approach to carry out the IPA. Steps 1–4 comprised an individual, inductive analysis with an iterative search for similarities and differences across emergent themes, leading to the development of superordinate and corresponding subordinate themes. Due to the inductive nature of IPA, the researchers carefully avoided applying transfer of training theory during part of the analysis of the individual in-depth interviews. Steps 5–6 involved performing a cross-case analysis, adding a layer of interpretation focusing on the perspective of transfer of training. This means that the results of the IPA were synthesized with the template analysis, leading to the emergence of the central themes. In accordance with the template analysis approach [23], a final hierarchical template was designed to ensure full inclusion of meaningful entities from the original dataset.

**Fig. 1:**
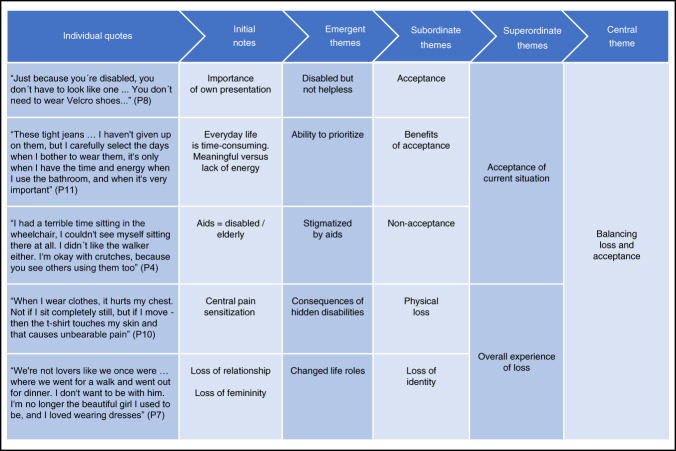
Example of a data-oriented audit trail. Illustration of the analytical process from the individual quotes to the development of the central theme: balancing loss and acceptance.

To enhance credibility of the analysis, the primary researchers (LW, NHV and TM) consistently discussed the analytical results until consensus was reached regarding emergent themes and the associated layers of interpretation. To ensure clinical relevance and enrich the interdisciplinary perspective, experts in SCI (FBS and NJH) and experienced qualitative researchers (LHS and recognized psychologist JM) took part in discussing the analytical results. Figure 1 presents an example of a data-oriented audit trail of the analytical process involving quotes reflecting the central theme.

## Results

### Participants

To reach a satisfactory sampling of 14 participants, the researchers contacted 17 selected individuals. One declined to participate, one was re-admitted to hospital, and one died. The interviews lasted 47 min to 2 h30 min (mean 1 h37 min). Table 1 presents participant characteristics. For reasons of anonymity, some data are presented in categories instead of as exact figures.

**Table 1:** Characteristics of participating individuals with SCI (*N* = 14).

Participant	Age (years)	Sex	Employment status	NLI^a^/AIS^b^	Mobility status	LOS^c^	Discharged^d^	Living arrangements
P1	31–45	Male	Sick leave	C1-C8 AIS D	Ambulatory	6–9	6–10	Cohabitating, with children
P2	16–30	Male	Student	T1-S5 AIS A, B, C	Wheelchair	6–9	6–10	Cohabitating
P3	61–75	Male	Retired	T1-S5 AIS D	Wheelchair	2–5	6–10	Alone
P4	46–60	Female	Sick leave	T1-S5 AIS D	Ambulatory with aids	2–5	2–5	Alone
P5	46–60	Male	Sick leave	T1-S5 AIS D	Wheelchair	6–9	2–5	Alone
P6	61–75	Female	Retired	T1-S5 AIS D	Ambulatory with aids	2–5	6–10	Alone
P7	31–45	Female	Sick leave	C1-C8 AIS D	Wheelchair	6–9	2–5	Cohabitating, with children
P8	16–30	Male	Student/part-time	T1-S5 AIS A, B, C	Wheelchair	6–9	6–10	Cohabitating, with parents
P9	61–75	Female	Retired	T1-S5 AIS D	Wheelchair	6–9	2–5	Alone
P10	31–45	Male	Sick leave	C1-C8 AIS D	Ambulatory	2–5	2–5	Cohabitating, with children
P11	16–30	Female	Sick leave	T1-S5 AIS A, B, C	Wheelchair	2–5	2–5	Alone
P12	31–45	Male	Part-time	T1-S5 AIS D	Ambulatory with aids	2–5	2–5	Cohabitating, with children
P13	46–60	Male	Sick leave	T1-S5 AIS D	Ambulatory with aids	2–5	2–5	Cohabitating, with children
P14	46–60	Female	Sick leave	C1-C8 AIS D	Wheelchair	6–9	2–5	Alone

^a^*NLI* neurological level of injury.

^b^*AIS* American Spinal Injury Association Impairment Scale.

^c^*LOS* length of stay at SCI unit (months).

^d^*Discharged* time since discharge (months).

### Key findings

**Fig. 2:**
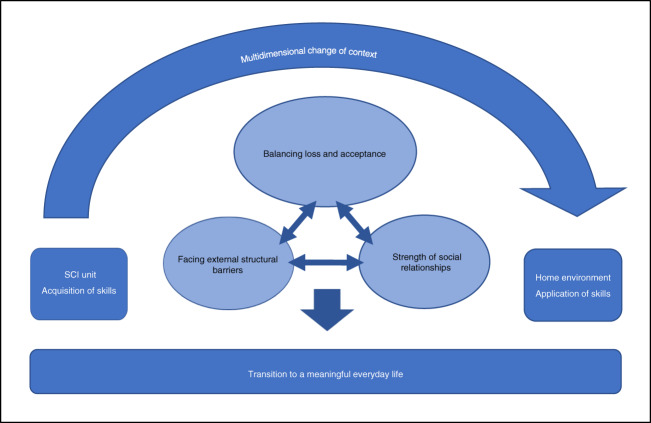
Summary of analytical results. The transition from the SCI unit to the home environment involves a multidimensional change of context. Within that overarching theme, three central themes are influencing the transition to a meaningful everyday life. Each theme has a mutual compensatory impact on the other, symbolized by reciprocal arrows.

Figure 2 summarizes the analytical results by capturing the overarching theme of multidimensional change of context in the transition from the SCI unit to the home environment. This dynamic model encompasses the three central themes that influence the transfer of knowledge and skills within the change of context: balancing loss and acceptance, facing external structural barriers, and the strength of social relationships, each of which have a mutual compensatory impact on the other. Supplementary online material (S2) presents the final underlying template for the figure.

### Overarching theme: multidimensional change of context

At the SCI unit, the participants consciously worked to acquire the skills they needed to manage the consequences of their injury. Their everyday lives were structured around targeted training and the introduction of a healthy lifestyle. In this context, ordinary life was inevitably put on hold by the absence of home, family, and work obligations (Table 2, quote (q) 1). At the SCI unit, a professional interdisciplinary team helped participants deal with the challenges they faced. The setting provided a peer group that made talking about complications such as altered bladder/bowel function socially acceptable (Tables 2, q2). Surrounded by an environment perfectly adapted to wheelchairs, the participants were better able to deal with their physical loss (Tables 2, q3). The unit in its entirety helped the participants maintain hope about their future level of functioning, allowing them to begin developing the satisfying feeling of regaining lost independence (Tables 2, q4).

**Table 2:** Participant quotes categorized under the overarching theme: multidimensional change of context.

Overarching theme: multidimensional change of context	
Quote no.	Contextual quotes
q^a^1	“My wife took care of our children and everything at home, so Hornbæk [SCI unit] was my sanctuary, where training was everything; it was more than a full-time job, even on weekends” (P^b^1).
q2	“At Hornbæk [SCI unit], we inspired each other and talked openly about our problems, even using the bathroom! We were all in the same boat, and although our injuries weren’t the same, we all had problems and could help each other” (P8).
q3	“Inspired by the spirit at Hornbæk [SCI unit]… the nurses very quickly urge, try to see if you can handle it on your own … and there’s an occupational therapist and physiotherapist teaching you some techniques and that’s how I continually got better” (P14).
q4	“My training at Hornbæk [SCI unit] was extremely satisfying. I felt insecure, but little by little I could handle things on my own because every single day I had training, and the staff supported me and helped me to challenge my boundaries” (P4).
q5	“The first three months back home were extremely difficult for me; I did almost nothing. I was uncertain and worried about the future, and working with the municipal authorities was stressful. It was the worst period since the injury” (P2).

^a^*q* quote.

^b^*P* participant.

**Fig. 3:**
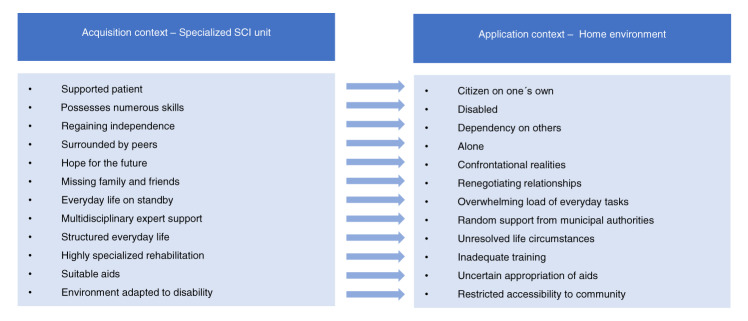
Interpretative summary of the experiences related to contextual change.

Transitioning to the home environment went far beyond the realm of a physical change in setting, as most of the participants’ previous life roles had changed. The participants with the most unresolved life circumstances described the transition as a bottomless pit with limited meaningfulness (Table 2, q5). In the participants’ homes, the interviewers observed that domestic tasks, left undone, were accumulating, with several participants apologizing for the lack of cleanliness. Figure 3 provides an interpretative summary of the experiences related to contextual change.

### Central theme 1: balancing loss and acceptance

Whether participants perceived their new everyday life as meaningful largely depended on how they experienced the consequences of SCI, which emerged as a balance between the experience of loss and acceptance of the current situation.

#### Experience of loss

The participants’ experience of physical loss was overwhelming in everyday life, some describing having lost their independence and sense of freedom. As the impact of SCI became visible in all aspects of everyday life, the physical loss evolved into a comprehensive loss of identity (Table 3, q6). At home, participants had to reconsider all social relationships, but changes in familial relationships were an especially sensitive area. Participants who were closely related to small children, either as parents or grandparents, experienced an extensive sense of deprivation in their relationship with the children. One participant with tetraplegia stated that being a mother left her with an overwhelming sense of inadequacy (Table 3, q7). In intimate relationships, a significant number of participants pointed out a loss of masculinity or femininity, not necessarily due to altered sexual function but in terms of how SCI negatively affected their caregiver role, ability to be a handyman, altered personal style, and use of health aids (Table 3, q8).

**Table 3:** Participant quotes categorized under central theme 1: balancing loss and acceptance.

Central theme 1: balancing loss and acceptance	
Quote no.	Experience of loss
q^a^6	“At Hornbæk [SCI unit], I was one of the healthy ones, or what do you call it… Someone who was fast and such… Now I’m back home; I am the disabled one. I’m having a hard time with that. Who am I now?” (P^b^10).
q7	“I don’t think my children deserve it… I just want to go for a walk with them or pick them up from daycare. I’ve such a bad conscience when I just turn on the TV, but it’s because you don’t have the same energy as before. I don’t feel like I’m teaching them anything. I’m not showing them how to live life” (P7).
q8	“I definitely feel less like a man, well… in every possible way. With the wheelchair, it’s my wife who carries me around. If we’re out shopping, she drags the heavy bags – and I used to take care of her; now she is taking care of me” (P10).

Quote no.	Acceptance of current situation
q9	“I knew I would never stand on my legs again, but I thought… does it matter? I can breathe, I can hug my family, I can say that I love them, and I can still play my PlayStation” (P8).
q10	“I love to cook and used to spend four hours making dinner on weekends. Now I’ve been recommended to buy as many kitchen appliances as possible and to use a machine to chop things instead. You must cheat and help yourself to save energy so that time is spent on the most important things” (P10).
q11	“I still enjoy my winter swimming club. A friend of mine rebuilt a wheelchair for me that resists water. Of course, I can’t jump into the sea, but the others shower me with two buckets of water and then I can join them in the sauna” (P5).
q12	“I dream of graduating and getting a job; that’s the big thing. Then I also have a goal that my boyfriend and I will be truly good for each other again… hopefully so good that one day we move back in together” (P11).

Quote no.	Risk of non-acceptance
q13	“You don’t want to sit in that wheelchair and live with that kind of pain every single day. You can’t live the same life as before; it’s just…. disabled… and I wasn’t BORN like that. I don’t have to accept it. I know what it feels like” (P7).
q14	“I have to admit that sometimes I try to hide that I have an injury, try to be… to look as normal as possible, try to get my old life back… that’s why training is the most important thing” (P1).

^a^*q* quote.

^b^*P* participant.

#### Acceptance of current situation

In this study, acceptance is defined as the *acceptance of the current situation*, based on the participants’ narrative of how they confronted the current consequences of SCI. A high level of acceptance helped to reduce the overall experience of loss but was not necessarily connected with a high level of physical functioning (Table 3, q9).

Participants with a high level of acceptance demonstrated characteristics that contributed to the application of skills in a changed, but still meaningful, daily life. The ability to prioritize the most important activities of the day was valuable. In this case, health aids were perceived and used as positive tools to achieve independence and to save time and energy (Table 3, q10). Participants were also able to define and preserve the essence of intrinsic values, despite altered physical function (Table 3, q11). Those with a high level of acceptance referred to themselves as disabled but not helpless. In addition, future dreams were based on reality and did not specifically depend on physical functioning (Table 3, q12).

#### Risk of non-acceptance

The five participants who predominantly experienced non-acceptance described feeling a sense of injustice and hopelessness, making it difficult to maintain a meaningful everyday life (Table 3, q13). These participants mentioned the family activities, jobs, and interests that they would initiate once they were 100% functional again. When an absence of meaning was prevalent, they failed to apply their acquired skills in everyday life. Participants with varying degrees of non-acceptance often perceived aids as stigmatizing. Everyday challenges were solved with an increased focus on improving physical functioning with the longing to be cured (Table 3, q14).

### Central theme 2: facing external structural barriers

#### Consequences of limited support

At home, all participants found the physical skills they had acquired meaningful, but only those who had wheelchair-adapted housing and who had been provided with the necessary aids successfully applied those skills. Most participants felt that collaboration with the municipal authorities was an additional burden. Navigating the bureaucracy of municipal institutions was demanding, and the request for aids and housing adjustments was rarely successful (Table 4, q15). For participants living alone and dependent on municipal home care, it was difficult to maintain a meaningful structure in everyday life when the required assistance lacked flexibility (Table 4, q16).

**Table 4:** Participant quotes categorized under central theme 2: facing external structural barriers, and central theme 3: strength of social relationships.

Central theme 2: facing external structural barriers	
Quote no.	Consequences of limited support
q^a^15	“The municipal authorities in my area… well, it felt like… whatever I did they kicked me when I was already down. There were problems with my training, and I’ve been fighting for a Batec [handbike] or a car and haven’t gotten either of them. How am I supposed to get back and forth from work?” (P^b^8).
q16	“They [home care] help me in the evening. Their arrival can vary up to an hour and a half, so I stopped having lunch, because then I have to go to the bathroom too early, and if they show up at 9 pm, it’s too late, then I’ve gone to the bathroom in my pants” (P3).
q17	“I’ve been referred to training twice a week, but that’s not enough. I know myself; I am an expert on my body. I have to be physically active EVERY DAY, so I feel like I’m alive, that I sense that I exist… So that I can DO something” (P7).

Quote no.	Healthy lifestyle
q18	“When I got home, I quickly found out how much effort ordinary things take. In the morning, I lie in bed and put on my clothes, but when I have to take a shower as well, then I almost need a nap again. It exhausts me when I decide to cook, and that’s why I started buying semi-prepared products” (P14).
q19	“The physical training offered by the municipality was disappointing, it lasted for three-quarters of an hour and most of it was waiting time. It was for the elderly, and there was a lot of talk about coffee and cake. So, there has been a long time where I haven’t trained, and I’ve also gained weight” (P8).
q20	“Now that I’m back home, there are SO many things I have to deal with. My kitchen is hopeless, my front door needs a ramp, I have to sell my car and then… job, girlfriend, and well… sports! I feel stressed, and I need to go to the doctor because I have high blood pressure” (P5).

Central theme 3: strength of social relationships	
Quote no.	Family support
q21	“My family… well, without them, I don’t think I would have come as far as I am today – and I certainly wouldn’t live my life if I sat alone in an apartment… my family give my life meaning” (P7).
q22	“Everything has changed. We used to help each other with household tasks. I feel guilty about that I just sit here, and she’s [wife] been at work all day, and then has to vacuum, cook, and do the laundry as well … but without my wife, my everyday life would be completely unmanageable” (P10).
q23	“My family thinks it’s ridiculous that I don’t want to catheterize myself lying on their couch, but I think it’s awful… I HATE IT when I lie there naked, with the fear of urine leaking, so I’d rather only be there for a short time” (P11).

Quote no.	Friendships
q24	“When someone comes to visit, it should be a positive experience. I think that’s one of the reasons my friends keep coming by, it’s because we enjoy each other’s company. Well… I can easily find something that makes me sad, but I want to be positive” (P5).
q25	“I am disappointed with many of my friends; they are not here for me when I need it most. This is a tragedy in my life, and I can’t bear to hear about their good lives, so I don’t pick up the phone when they call” (P7).

^a^*q* quote.

^b^*P* participant.

To support the use of acquired skills, all participants were discharged from the SCI unit with a plan for further training in a municipal setting. Two-thirds of participants described the continued training as short-lived, lacking appropriate equipment, and as suffering from a scarcity of SCI expertise. One participant stated that inadequate training led to reduced arm strength and a poorer ability to execute wheelchair transfers, which he perceived as a possible cause of his pressure ulcer. Participants were also mentally affected by the inadequate training, as training provided hope for future functioning and brought a sense of meaningfulness to their day (Table 4, q17).

#### Healthy lifestyle

Healthy living was perceived as a balance of healthy meals and increased exercise, but participants experienced everyday tasks as overwhelming and time-consuming. For example, painful hands and fingers, inadequate access to shopping, reduced energy, and lack of time were obstacles to preparing healthy meals (Table 4, q18). The exercise provided by the municipal authorities, which did not specifically support cardiovascular training, made it difficult to continue the healthy lifestyle introduced at the SCI unit (Table 4, q19). Lifestyle changes were also perceived as a distant project that, due to everyday challenges, could not be given high priority in the early stages of transition (Table 4, q20).

### Central theme 3: strength of social relationships

#### Family support

The emotional narratives of the participants made it clear that maintaining close relationships was crucial, but also a sensitive topic. Family and friends were a strong motivating factor for improving physical functioning and greatly enabled the application of skills at home. Participants who had close relationships described them as extremely valuable for obtaining a meaningful everyday life (Table 4, q21).

For some participants, the significant impact on relationships became particularly evident in the confrontation with their post-discharge reality. Maintaining partnerships was a major challenge, and two relationships did not survive the transition to home. Particularly challenging was the participants’ increased dependency on others. The need for assistance led to alternating between feeling guilty and feeling gratitude toward spouses and family members who took responsibility for solving most practical challenges (Table 4, q22). Strong relationships were often associated with meaningful activities outside the home, although some aspects of care could also be frustrating and lead to the avoidance of social activities (Table 4, q23).

#### Friendships

For the majority, it was essential to maintain previous friendships. Restoring friendships post-discharge was challenged by the absence of joint activities and reduced reciprocity. Participants who were able to maintain friendships succeeded, along with their friends, in redefining shared values. With humor and openness, participants tried to eliminate SCI taboos and to focus on the positive aspects of life (Table 4, q24).

Participants with a high degree of non-acceptance often experienced a lack of understanding and support from their friends and found it difficult to relate to their friends’ current lives. These participants said that they felt like they were fighting a lonely battle and that using their skills, especially outside the home, was limited (Table 4, q25).

## Discussion

This study contributes to furthering understanding of the perception of transferring knowledge and skills to the home environment and identified the multidimensional change of context present when the SCI unit’s safe and supportive environment was replaced by physical, emotional, and social uncertainty at home.

To understand contextual challenges, our study addressed the perspective of transfer of training, as described by Baldwin and Ford [16] and elaborated on by Grossman and Salas [17]. The authors emphasized that successful transfer of skills required similarity between the acquisition context (in this study, the SCI unit) and the application context (home environment). They also indicated that successful transfer outcomes heavily depend on the application context, e.g., having access to the necessary equipment, using skills in direct extension of learning, and strong social support [17]. The present study and other literature [13–15] indicate that there are remarkable differences between the supportive environment found in SCI units and everyday life limitations in the home, which may partially explain why the transition between the two is difficult. Nunnerley et al. [14] proposed that SCI rehabilitation does not focus on dealing with the real world. The SCI unit in our study highly prioritized preparing people for everyday life by incorporating home visits during inpatient rehabilitation. Despite this, the transition to home was perceived as extremely difficult because most life roles had changed, making social renegotiation necessary.

In the present study, attempts to continue a healthy lifestyle post-discharge may have faced obstacles because the acquisition and application contexts lacked identical elements in terms of making meals and exercise. Without supportive structures post-discharge, there was a limited agenda for managing cardio-metabolic risk after SCI [9]. Randomized controlled trials examining the effectiveness of lifestyle interventions have conflicting findings [24, 25]. Nooijen et al., whose study is the most promising, found that an intervention initiated during inpatient rehabilitation coupled with additional behavioral intervention and a visit to optimize the home environment was effective in eliciting behavioral change toward a more active lifestyle [25]. Participants in the present study, however, perceived lifestyle changes as an additional burden rather than as a solution to everyday challenges, indicating that tertiary health promotion may still be a distant prospect.

Our study indicates that transferring knowledge and skills into a meaningful everyday life required an individual balancing of loss and acceptance. Tulsky et al. [26] confirmed the importance of managing loss and found that, in terms of emotional health, managing loss and grief was of the highest priority. Within SCI research, awareness of acceptance is increasing. Aaby et al. [27] emphasized that acceptance is a psychological resource that healthcare professionals can work to support to improve quality of life and mental health following SCI. Incorporating Stroebe and Schut’s dual process model of coping with bereavement can aid in understanding how loss and acceptance relate to transfer of knowledge and skills [28]. Central to their model is the necessity of accepting the reality of the changed world. The authors concluded that processing grief encompassed alternating between loss orientation involving grief work and restoration orientation, including taking time off from the pain of grief and attending to one’s life changes. Translating this theory into the present findings, we propose that the SCI unit setting, which offers a wheelchair-adapted environment, interdisciplinary support, and social encouragement supports the alternation between grief over lost physical function and acceptance of a changed everyday life. However, returning home to an environment with limited physical opportunities and social insecurity may reinforce the grief of having sustained a SCI, causing loss orientation to become more prominent.

Moreover, our findings suggest that when individuals with SCI are left on their own to navigate in a municipal system that does not prioritize and coordinate their needs, unnecessary external pressure is imposed on them, which may partially contribute to the psychological burden that individuals with SCI experience [3, 4]. We propose that individuals with SCI might benefit from a municipal coordinator to support navigation between the various actors in the municipality.

Successful transfer of knowledge and skills was mediated by valuable social relationships, both in terms of close relatives and friendships. When social relationships were still enriching post-discharge, they added meaningfulness and contributed to the acceptance of a changed everyday life. These results are consistent with findings from a systematic review conducted by Muller et al. [29], who concluded that social support was associated with higher quality of life, lower morbidity and mortality, and increased community participation among individuals with SCI. In line with this, we found that strong support from family or friends enabled the participants to apply their acquired skills in meaningful activities outside the home. Maintaining this social support was difficult, and re-establishing relationships required extraordinary personal resources. The psychosocial support that the SCI unit provided was valuable, but participants only truly encountered, and had to manage, the full extent of the social consequences of their injury in their home environment. Moreover, close relatives handled most everyday tasks at home, putting family support at high risk of changing from being a positive factor to becoming a burden. Recent knowledge about the caregiver burden in terms of SCI has shown that it can be stressful [30]. In line with transfer of training theory, continuity of skills training and access to community services have been shown to contribute to the sustainability of the family caregiving role [30].

Our study emphasizes the necessity of bridging the gap between the SCI unit and the home environment to support physical and emotional concerns and meaningful relationships. It is essential that healthcare professionals engage in open dialog concerning transitional challenges and support the life areas that are most meaningful to the individual. Interventional research might explore the value of a gradual discharge with, e.g., the establishment of an outgoing specialized team. This interdisciplinary team might advise and educate municipal stakeholders to support adequate exercise and care opportunities in the home environment.

### Strengths and limitations

The participants in this qualitative study had a desire and comprehensive need to communicate their experiences, providing a substantial amount of information and sufficient saturation to address the research question. The transparent application of two complementary qualitative methodologies raised the researchers’ reflexivity during the interpretation phase. Interviews were conducted in the participants’ homes, which allowed for direct observation and acknowledgment of everyday challenges. The sample reflected variation according to age, sex, family relationships, and level of injury. However, it is unknown whether the analysis is adequate for individuals with complete tetraplegia as they represent a proportionally small group in our SCI unit and none of them were in the study population. Our data and conclusions are limited to the early stage of transition. This study does not provide the foundation to determine whether immediate success or difficulties post-discharge would produce the same trend regarding long-term community integration.

## Conclusion

Transition from the SCI unit to the home environment is influenced by a multidimensional change of context and the need for continuity in the reestablishment of a meaningful everyday life is striking. Limited opportunities in the home environment may restrict the use of acquired skills post-discharge, provide distant prospects for tertiary health promotion, and aggravate the experience of loss in people with SCI. Maintaining relationships is a strong mediator for transferring skills and the acceptance of a changed everyday life. Managing transitional difficulties is relevant in cross-sectoral SCI rehabilitation and findings from this study can be incorporated in interventional research aimed at the early stages of transition and long-term community reintegration.

## Supplementary information

Supplemental material (S1) Interview guide and Supplemental material (S2) Final template

## Data Availability

The datasets generated and analyzed in the current study are not publicly available due to the possibility of identifying information in the in-depth qualitative data, but can be made available from the corresponding author upon reasonable request.
