# Supplementary material for: Exploring the contextual transition from spinal cord injury rehabilitation to the home environment: a qualitative study
Source: Spinal Cord. 2021 Feb 9;59(3):336–46. doi: 10.1038/s41393-020-00608-y (PMC7943422; doi:10.1038/s41393-020-00608-y)
Supplement: Supplementary file 1 — Supplemental material (S1) Interview guide and Supplemental material (S2) Final template [file 41393_2020_608_MOESM1_ESM.pdf]

Spinal Cord, 2020

Exploring the contextual transition from spinal cord injury rehabilitation to the home environment: a qualitative study

Author information:
Lene Weber, Nanna Hoffgaard Voldsgaard, Nicolaj Jersild Holm, Lone Helle Schou, Fin Biering-Sørensen, Tom Møller

Supplemental material (S1) presents the complete interview guide, which also represents the initial template.

Supplemental material (S1) Interview guide

A priori themes	Codes	Examples of open-ended questions
The individual story	Story of the SCI	Tell me about yourself and why you were admitted to the SCI unit?
Experience of specialized rehabilitation	Acquisition of skills	Were any parts of the rehabilitation particularly meaningful to you?
	Goals and motivation	What goals did you set for your rehabilitation?<br>What motivated you?
	Weekend leave	How did you experience weekend leave?
	Discharge	How did you prepare for discharge?
Experience of everyday life	Transitioning home	Can you tell me about your everyday life?
	Applying skills	How do you apply the skills you learned at the SCI unit now that you are at home?
	Lifestyle	What thoughts do you have about healthy living? How does it unfold in your everyday life?
	Impact of SCI<br>(physical, emotional and social)	How has the SCI affected your relationship with family and friends?
	Structural support	How do you experience the collaboration with your municipality?
	Future	What thoughts do you have about the future?

Abbreviation: SCI, spinal cord injury

Spinal Cord, 2020

Exploring the contextual transition from spinal cord injury rehabilitation to the home environment: a qualitative study

Author information:
Lene Weber, Nanna Hoffgaard Voldsgaard, Nicolaj Jersild Holm, Lone Helle Schou, Fin Biering-Sørensen, Tom Møller

Supplemental material (S2) presents the final template, which is based on the systematic text analysis as the underlying foundation of the illustration in Fig. 2.

Supplemental material (S2) Final template

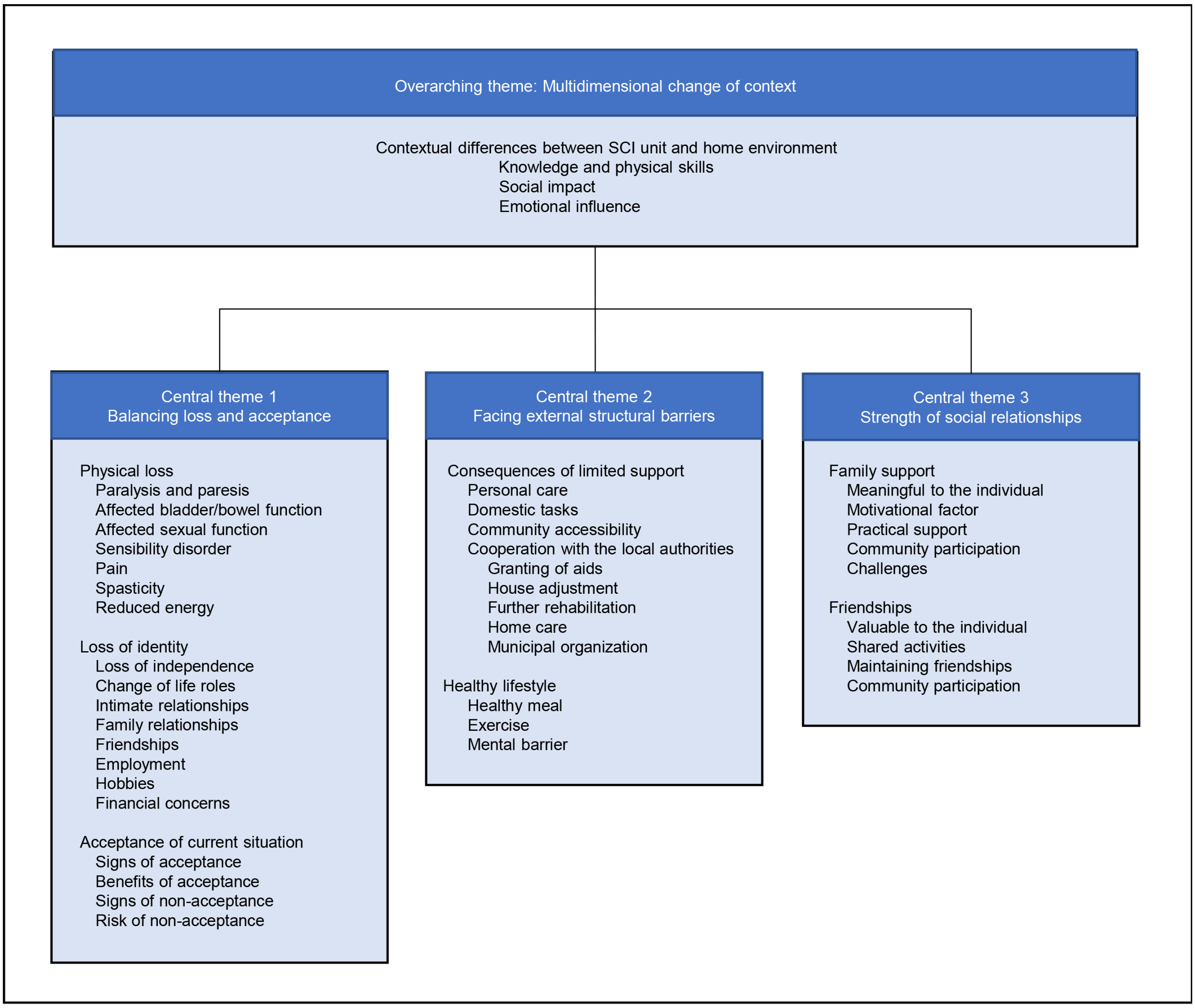

Abbreviation: SCI, spinal cord injury
